# The Mammalian High Mobility Group Protein AT-Hook 2 (HMGA2): Biochemical and Biophysical Properties, and Its Association with Adipogenesis

**DOI:** 10.3390/ijms21103710

**Published:** 2020-05-25

**Authors:** Linjia Su, Zifang Deng, Fenfei Leng

**Affiliations:** Biomolecular Sciences Institute, Department of Chemistry & Biochemistry, Florida International University, Miami, FL 33199, USA; lsu008@fiu.edu (L.S.); zdeng004@fiu.edu (Z.D.)

**Keywords:** HMGA2, AT-hook, adipogenesis, intrinsically disordered protein, stem cell

## Abstract

The mammalian high-mobility-group protein AT-hook 2 (HMGA2) is a small DNA-binding protein and consists of three “AT-hook” DNA-binding motifs and a negatively charged C-terminal motif. It is a multifunctional nuclear protein directly linked to obesity, human height, stem cell youth, human intelligence, and tumorigenesis. Biochemical and biophysical studies showed that HMGA2 is an intrinsically disordered protein (IDP) and could form homodimers in aqueous buffer solution. The “AT-hook” DNA-binding motifs specifically bind to the minor groove of AT-rich DNA sequences and induce DNA-bending. HMGA2 plays an important role in adipogenesis most likely through stimulating the proliferative expansion of preadipocytes and also through regulating the expression of transcriptional factor Peroxisome proliferator-activated receptor γ (PPARγ) at the clonal expansion step from preadipocytes to adipocytes. Current evidence suggests that a main function of HMGA2 is to maintain stemness and renewal capacity of stem cells by which HMGA2 binds to chromosome and lock chromosome into a specific state, to allow the human embryonic stem cells to maintain their stem cell potency. Due to the importance of HMGA2 in adipogenesis and tumorigenesis, HMGA2 is considered a potential therapeutic target for anticancer and anti-obesity drugs. Efforts are taken to identify inhibitors targeting HMGA2.

## 1. Introduction

The mammalian high-mobility-group protein AT-hook 2 (HMGA2) is a non-histone chromosome protein and belongs to the HMGA family, which includes four members: HMGA1a, 1b, 1c, and HMGA2 [1]. HMGA1a, 1b, and 1c are the different splicing products of the same gene, the HMGA1 gene [2]. HMGA2 is the product of a different gene, the HMGA2 gene [3,4,5]. High-mobility-group proteins were discovered, identified, and isolated by Graham H. Goodwin in E. W. Johns’ lab at Chester Beatty Research Institute, UK, in the early 1970s [6,7]. High-mobility-group (HMG) proteins simply refer to the group of fast migration, non-histone proteins in the polyacrylamide gels when calf thymus chromatin was extracted using 0.35 M NaCl and 2% trichloroacetic acid [6,7]. Initially, only two HMG protein families, i.e., HMGB protein family (HMG-box proteins; former name HMG1/2 proteins [8]) and HMGN protein family (nucleosome binding proteins; former name HMG-14/17 proteins [8]) were identified [9]. HMGA1a/1b (former name HMG-I/Y [8]) were identified in 1983 in Hela S3 cells by Lund et al. [10]. HMGA2 (former name HMGI-C [8]) was discovered in 1985 by two different groups, Vincenzo Giancotti’s group at Universita di Trieste, Italy [11], and Graham H. Goodwin’ group, in the UK [12]. Interestingly, HMGA2 was only expressed in virus-transformed cells [11,12]. The cDNA sequence and protein sequence of murine and human HMGA2 were published in 1991 [3] and 1994 [13], respectively. The protein sequences of murine and human HMGA2 are almost identical, except for five amino acid residues. None of these five amino acid residues is located in the “AT-hook” DNA-binding motifs [3,13]. The human HMGA2 gene is located at chromosome 12, 12q14.3 [14,15], and has five exons and four introns, occupying approximately 160 kb [16]. Intron 3 is very large ~110 kb [5] and separates the “AT-hook” DNA-binding motifs and the acidic C-terminus [16]. The 4.1 kb mRNA contains an 854 bp 5′ UTR, a 330 bp coding sequence, and a 2966 bp 3′ UTR [16]. The 3′ UTR carries multiple microRNA Let-7 binding sites that negatively regulate HMGA2 expression in development and tumorigenesis [17,18,19].

## 2. Biochemical and Biophysical Properties of HMGA2

**Figure 1:**
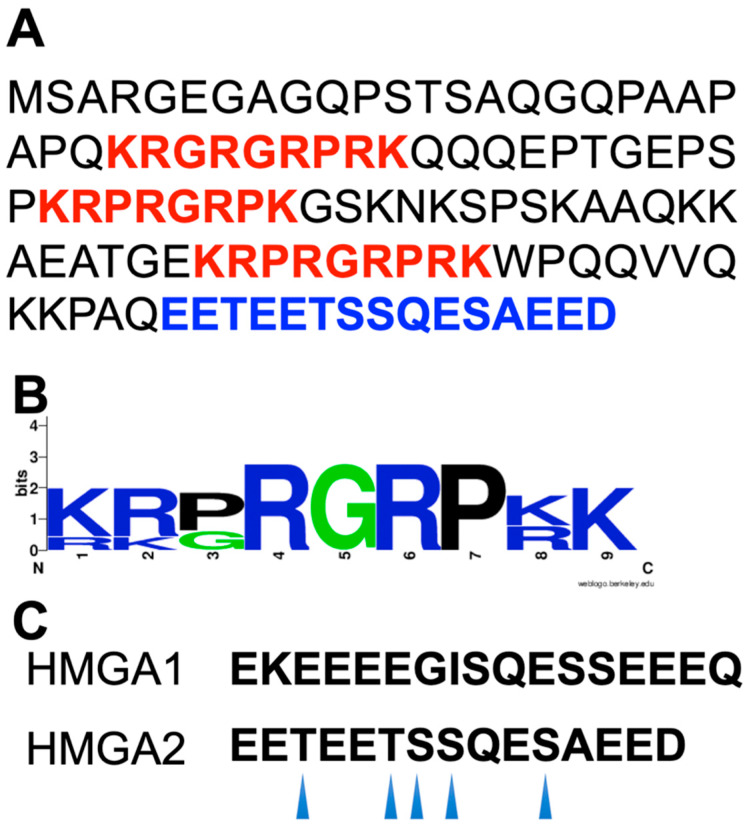
(**A**) The primary structure of the human HMGA2. The positively charged “AT-hook” DNA binding motifs and the negatively charged C-terminal motif are highlighted in red and blue, respectively. (**B**) Sequence logo of the “AT-hook” DNA-binding motifs of HMGA1 and HMGA2. Sequence conservation, measured in bits of information, is illustrated by the height of stacking. The sequence logo was generated by WebLogo (available at https://weblogo.berkeley.edu/logo.cgi). (**C**) The C-terminal motifs of HMGA1 and HMGA2. The CK2 phosphorylation sites of the HMGA2 C-terminal motif are indicated by arrows.

The human HMGA2 is a small DNA-binding protein and has 109 amino acid residues (Figure 1). One unique feature of HMGA2 is the asymmetric charge distribution along its backbone (Figure 1). As a consequence, HMGA2 can form homodimers in aqueous buffer solution [20]. Early studies also showed that HMGA2 forms dimers, trimers, and tetramers, although it was attributed to the formation of a disulfide bond between the cysteine (Cys) residues of murine HMGA2 (murine HMGA2 has a Cys reside at position 41) [21]. Nevertheless, the formation of trimers and tetramers cannot be explained by the disulfide-bond formation. A different study also demonstrated that HMGA1a could interact with itself [22]. The dimerization of HMGA proteins is an unusual property because HMGA proteins, including HMGA1 and HMGA2, are intrinsically disordered/unstructured proteins (IDPs) [20]. In other words, this family of proteins does not have a secondary structure and a tertiary structure; however it has a quaternary structure. It was initially quite a challenge to publish our results by showing that HMGA2 can form homodimers and homo-oligomers in aqueous buffer solution, although this unique feature of HMGA2 was observed in the early 2000s [23]. Nevertheless, biochemical and biophysical studies clearly demonstrated that HMGA2 can form homodimers [20]. Of course, HMGA2 is not the only IDP that can form homodimers; other IDPs can also form homodimers [24,25,26,27,28,29]. The cytoplasmic region of T-cell receptor subunit and the disordered N-terminal domain of ultraspirale from Aedes aegypti (aaUsp-NTD) can self-associate into homodimers [26]. Intriguingly, the dimerization is not accompanied by a disorder-to-order transition [26]. Although several IDPs can self-associate into homodimers and/or homo-oligomers, two important questions are still unanswered: (1) Can one IDP interact with another IDP? The homodimerization and oligomerization of IDPs partially answered this question. (2) What forces contribute to the interaction between IDPs? As we discussed above, the dimerization of HMGA2 mainly stems from the electrostatic interactions between the positively charged “AT-hooks” and the negatively charged C-terminus, since the asymmetric charge distribution is along the HMGA2 backbone. Are hydrophobic force and hydrogen bonds also involved in the dimerization?

**Figure 2:**
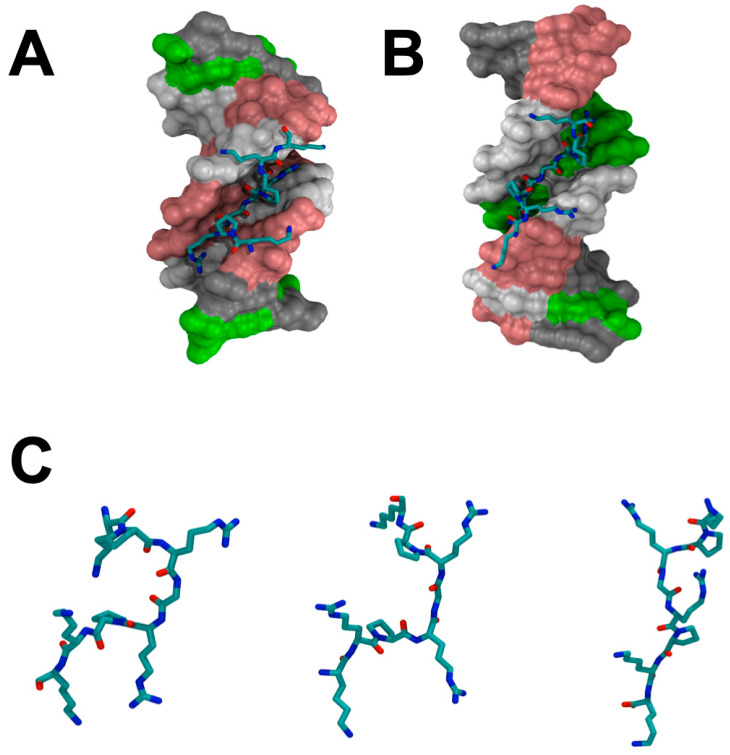
(**A**) The crystal structure of an “AT-hook” and DNA complex [33]. (**B**) The solution NMR structure of the complex of an “AT-hook” DNA-binding motif with DNA determined by Huth et al. [32]. (**C**) Comparison of the “AT-hook” DNA-binding motifs from the crystal structure, the NMR solution structure, and the simulation structure. The molecular dynamic simulation was performed by using NAMD with CHARMM36m force field [36,37,38] for 20 ns, 2 fs/time step, 310K, and 12.0 Å VDW force cutoff.

Another unique feature of HMGA proteins is that all except HMGA1c contain three “AT-hook” DNA-binding motifs (Figure 1). The “AT-hook” DNA-binding motif is an 8-9 amino acid peptide that contains 5-6 positively charged amino acid residues, lysine and arginine (Figure 1). Specifically, this DNA-binding motif has a consensus palindromic sequence, PRGRP surrounded by one or two positively charged amino acid residues (Figure 1). The “AT-hook” DNA-binding motif was coined by Reeves and Nissen [30]. They demonstrated that the conformation of this consensus DNA-binding motif is similar to several typical DNA minor groove binders, such as netropsin, distamycin, and Hoeshst33258, and can preferentially bind to minor grove of AT-rich DNA sequences [30]. Indeed, nuclear magnetic resonance (NMR) and crystal structural studies showed that the “AT-hook” DNA-binding peptide specifically binds to the minor groove of AT-rich DNA [31,32,33] (Figure 2). NMR and crystal structures are quite similar with the central RGR group deeply penetrating into the minor groove of AT base pairs [32,33]. The crystal structural study showed that the “AT-hook” also forms hydrogen bonds between the backbone NH groups of the peptide and the thymine in the minor groove [33]. It was also discovered that the DNA is bent and the minor groove is widened [33]. The HMGA2-induced DNA bending was also observed when gel permutation assay was used [34]. The HMGA2-induced bending angle was determined to be 35 degrees, which was significantly larger than the one (24 degrees) observed in the crystal structure induced by just one “AT-hook” DNA-binding motif, suggesting that more than one “AT-hook” was involved in the DNA binding and bending [34]. Although it was suggested that “AT-hook” DNA-binding motifs adopt a defined structure upon binding to AT-rich DNA sequences, recent studies and molecular simulations do not support a disordered-to-ordered structural transition of the “AT-hook” DNA-binding motif upon DNA binding (Figure 2B) [35].

Early DNA foot-printing studies showed that HMGA proteins could bind to any stretches of 5 to 6 AT bp with similar binding affinities [39], suggesting that binding of these proteins to AT-rich DNA sequences does not have sequence specificities. However, other studies demonstrated that HMGA proteins prefer binding to two-to-three appropriately spaced AT-rich DNA sequences with high DNA-binding affinities [40]. More importantly, HMGA proteins bind to two to three runs of AT base pairs in the promoter regions, as a transcription factor to regulate transcription in vivo [41,42,43,44]. NMR and crystal structural studies also showed that “AT-hook” DNA-binding motifs prefer certain AT DNA sequences [32,33]. Encouraged by these results, we performed a PCR-based systematic evolution of ligands by exponential enrichment (SELEX) experiment and identified two consensus DNA-binding sequences for HMGA2, 5′ATATTCGCGAWWATT-3′, and 5′-ATATTGCGCAWWATT-3′, where W represents A or T [45]. This is an interesting result in that the HMGA2 preferred binding sequences contain four GC base pairs in the middle [45]. Since the minor groove of GC base pairs is crowded, it is likely that not all three “AT-hook” DNA-binding motifs bind to the DNA minor groove. Possibly one of the “AT-hook” DNA-binding motifs binds to the major groove of the middle GC-rich DNA sequence. Our recent results showed that the “AT-hook” DNA-binding motif could indeed bind to the DNA major groove (unpublished results). ChIP experiments using cancer cells overexpressing HMGA2 showed that HMGA2 prefers binding to AT-rich DNA sequences, although the center sequences are not necessarily GC-rich [46]. 

Another unique feature of HMGA proteins is that all contain a highly negatively charged C-terminal motif. For instance, HMGA2 has a 15 amino acid residue C-terminus, with seven glutamic acid residues and one aspartic acid residue (Figure 1). The C-terminus also contains three serine residues and two threonine residues that can be phosphorylated by casein kinase 2 (CK2) [47,48]. If fully phosphorylated, the C-terminus of HMGA2 may carry up to 19 negative charges at physiological conditions (each phosphate group introduces two negative charges). Since the electrostatic interaction is an important force for the binding of HMGA2 to AT-rich DNA [49], one possible function of the C-terminus of HMGA proteins is to regulate the DNA-binding affinity during different cellular events. Indeed, previous results showed that the negatively charged C-terminus and its phosphorylation could regulate the DNA-binding capacity of HMGA proteins [47,48,50,51,52]. The C-terminal motif of HMGA proteins may also be involved in the protein–protein interactions. The truncated HMGA2 without the C-terminal motif cannot form a homodimer [20]. The C-terminal motif may also be involved in the interaction of HMGA proteins with its protein partners [22,53,54]. Nevertheless, the biological functions of the C-terminal motif are still unknown, although it was implied that it might contribute to the tumorigenesis and cellular proliferation and transformation [43,55].

## 3. HMGA2 in Adipogenesis

The association of HMGA2 with adipogenesis was discovered by Chada and coworkers when they studied mouse growth-hormone-independent pygmy phenotype in the early 1990s [56,57,58]. They showed that this mouse pygmy phenotype stems from the deletion of mouse Hmga2 gene from the chromosome, and, as a result, HMGA2 was not expressed during embryogenesis [58]. Further, they demonstrated that HMGA2 only expressed in early embryonic stage from 10.5 to 15.5 d.p.c. (days post-coitum) and did not express in mouse adult tissues [58]. They also showed that HMGA1 was predominantly expressed in 10.5–16.5 d.p.c. mouse embryos [58]. By analyzing 11.5 d.p.c. mouse embryos, they found that HMGA2 expression was observed in most tissue and organs except the brain. Only a small localized region of forebrain had HMGA2 expression [58]. The testes and adrenal gland of the mutant mice are much smaller [57,59]. In fact, Hmga2 null mice are sterile due to the fact that germ-cell maturation was blocked in the testes [57,59]. Nevertheless, the most noticeable phenotype of the Hmga2 deletion mice is the small size. At 10 weeks of age, the body weight of Hmga2 mutants is approximately 40% of that of the wild-type mice [57,59]. These mutant mice have significantly reduced body fat compared to the wild-type mice [57,59]. Additionally, the mutant mice are resistant to a high-fat diet [57,59]. In contrast, a high-fat diet can induce the HMGA2 expression in adipose tissues and cause obesity in wild-type and leptin-deficient mice [60]. They performed an interesting experiment by using the genetic mouse model Lep^ob^/Lep^ob^ to generate two mouse models: Hmga2^-/-^ Lep^ob^/Lep^ob^ and Hmga2^+/-^ Lep^ob^/Lep^ob^ [60]. The disruption of the Hmga2 gene caused a dramatic reduction in obesity of the leptin-deficient mice (Lep^ob^/Lep^ob^) in a gene-dosage-dependent manner: Hmga2^+/+^ Lep^ob^/Lep^ob^ mice weighed over three times more than Hmga2^-/-^ Lep^ob^/Lep^ob^ animals, and the weight of Hmga2^+/-^ Lep^ob^/Lep^ob^ mice was in between [60]. The adipocytes of the mutant mice are similar to those of the wild-type mice, and the expression levels and regulations of genes involved in adipogenesis are also similar [60]. The reduction of body fat is a result of a decrease of the cell numbers in the adipocyte tissues [60]. More recently, Federico et al. created Hmga1 and Hmga2 double-knockout mice that have a “superpygmy” phenotype, with 75% smaller size than that of the wild-type mice [61]. The body fat should also be greatly reduced. Several studies with transgenic mice overexpressing HMGA2 also demonstrated the association of HMGA2 with adipogenesis [55,62,63]. For instance, Battista et al. created a transgenic mouse model that expresses a truncated HMGA2 carrying 3 “AT-hook” DNA-binding motifs without the acidic C-terminal motif. These transgenic mice developed a giant and obese phenotype [55] with a great expansion of adipocyte tissues. In additional to a great enhancement of abdominal fat mass, large fat pads were also associated with other organs, such as around the kidneys and at the bases of the hearts [55].

More evidence of HMGA2’s association with adipogenesis comes from the studies of Lipomas, which are a type of benign tumor that is made of fat/adipocyte tissues and often found with the chromosomal arrangement at 12q14-15 [14,15]. Early studies showed that these common mesenchymal neoplasms resulted from the expression of a chimeric protein consisting of the three “AT-hook” DNA-binding motifs fused to LIM or an acidic transactivation domain [14,15,64]. Further studies showed that the expression of the three “AT-hook” DNA-binding motifs alone is sufficient for the formation of lipomas [55,63,65]. In 2005, a case was reported for an eight-year-old boy who has a phenotype of overgrowth, advanced endochondral bone, a cerebellar tumor, and multiple lipomas [66]. Molecular analyses showed that this abnormal phenotype stems from the inversion of chromosome 12, with breakpoints at p11.22 and q14.3 that resulted in the expression of a truncated HMGA2 only with the three “AT-hook” DNA-binding motifs and lacking the negatively charged C-terminal motif [66]. This phenotype is similar to that of transgenic mice described above [55]. Genome-wide association (GWA) studies using single nucleotide polymorphism (SNP) data found that HMGA2 is associated with human height in the general population across different ethnicities or races [67,68,69,70,71,72,73,74]. Specifically, several SNPs, such as rs1042725 and rs10784502, located in the 3’ UTR of HMGA2 gene, are associated with human height [67,75]. Surprisingly, rs10784502 was also found to associate with human intracranial volumes and intelligence quotient (IQ) [76]. The association of HMGA2 with human height was further demonstrated by 12q14 microdeletion syndromes in which several genes, including HMGA2, were deleted [77,78]. One common phenotype is the short stature and growth failure [77,78]. For example, case #D0811079 is a boy who has a deletion that only involved HMGA2. Besides the short stature, no other anomalies were observed for this patient [78].

Adipocytes are derived from multipotent mesenchymal stem cells (MSCs) through two distinction phases: the commitment of MSCs to preadipocytes and the differentiation of preadipocytes to mature adipocytes [79,80,81,82,83]. The route of MSCs to preadipocytes is quite complex and can be driven by different signaling pathways [81,82]. For the differentiation pathway of preadipocytes to mature adipocytes, two steps are involved: clonal expansion and adipocyte maturation [81,82]. Our understanding of the molecular mechanism of preadipocytes differentiation into mature adipocytes mainly came from the studies of model preadipocyte cell lines that are committed to differentiating into adipocytes, such as 3T3-L1 and 3T3-F422A [81,84,85]. Several transcriptional factors, such as CREB (cAMP response element-binding) protein, CEBPβ (CCAAT/enhancer binding protein β), CEBPα, and PPARγ (peroxisome proliferator-activated receptor γ), are involved in this process [79,80,81,82,83]. Recent studies showed that HMGA2 is highly expressed during the exponential growth of 3T3-L1 cells [86]. Its expression is significantly reduced upon growing to confluence (the quiescent state; [86]). Interestingly, after the addition of differentiation cocktail, HMGA2’s expression is induced again and reaches the highest level after two days (the mRNA reaches the highest level after a six-to-eight-hour induction [86,87,88,89,90]. HMGA2 graduate decreases to the basal level after the cells are differentiated into mature adipocytes [86,87,88,89,90]. Current evidence showed that HMGA2 functions at the clonal expansion step and regulates the expression of transcriptional factor PPARγ [90]. HMGA2 expression level is also negatively regulated by microRNA let-7 [88], a factor that plays critical role in stem cells’ self-renewal and stemness [17,18,19]. Some other micro RNA species, such as microRNA 33b, may also be involved in this process [91]. Although more studies are needed to determine the molecular mechanism of HMGA2 for adipogenesis, it is likely that HMGA2 affects adipogenesis through a mechanism similar to that through which it regulates other stem cells and their differentiation. Below, we briefly review HMGA2’s association with cell youth and self-renewal of stem cells [92,93,94,95], one of its most intriguing functions.

As shown above, mouse HMGA2 only expressed in early embryonic stage and did not express in the adult tissues [58]. Similar to mouse HMGA2 expression pattern, HMGA2 was expressed in all human fetal tissues [96,97]. In contrast, HMGA2 did not express in most adult tissues, except for lung and kidney [97]. These results suggest that HMGA2 is mainly expressed during embryonic and fetal development. Interestingly, HMGA2 is highly expressed in human stem cells, including human embryonic stem (hES) cells and the early differentiating embryoid bodies (EBs) [98,99]. For instance, Nishino et al. showed that HMGA2 expression is highly expressed in neural stem cells (NSCs) and declines with age [100]. This decrease is partially caused by the increasing expression of a microRNA let-7b that targets the 3′ UTR of Hmga2 mRNA [100]. They further demonstrated that HMGA2 promotes NSC self-renewal in young, but not in old, mice, most likely through a new pathway by which HMGA2 expression was inhibited by let-7b. As a result, JunB and P16Ink4a/P19Arf expression was enhanced [100]. These results are consistent with an earlier study showing that let-7 regulates self-renewal and stemness of breast cancer stem cells [18]. Since then, HMGA2 was shown to link to the stem cell youth and self-renewal of other stem cells and progenitors [101,102,103,104,105,106,107,108,109]. For example, the self-renewal capacity and youth of hematopoietic stem cells (HSCs) is linked to expression of HMGA2 [103]. It was also demonstrated that the expression of HMGA2 was able to rescue the in vitro aging process of mesenchymal stem cells [110]. The self-renewal potential/capacity is determined by a unique pathway involving the RNA-binding protein Lin28, the microRNA let-7b, and HMGA2, in which Lin28 binds to let-7 pre-microRNA and inhibits the generation of let-7 [103,109]. In 2010, Cavazzana-Calvo et al. reported a case of successful gene therapy of human β-thalassemia, a genetic disease with mutations in the β-globin gene that reduce or abolish β-globin protein production [111]. An adult patient with severe β^E^/β^0^-thalassaemia who was dependent on monthly transfusion became transfusion-independent after receiving the lentiviral-based gene therapy where the modified HSCs with β-globin lentiviral vector were transplanted into the patient’s bone morrow [111]. Surprisingly, the therapeutic efficacy stems from the overexpression of HMGA2 in HSCs or progenitor cells to produce nucleated blood cells with overexpressed HMGA2 [111].

## 4. Conclusions and Perspectives

**Figure 3:**
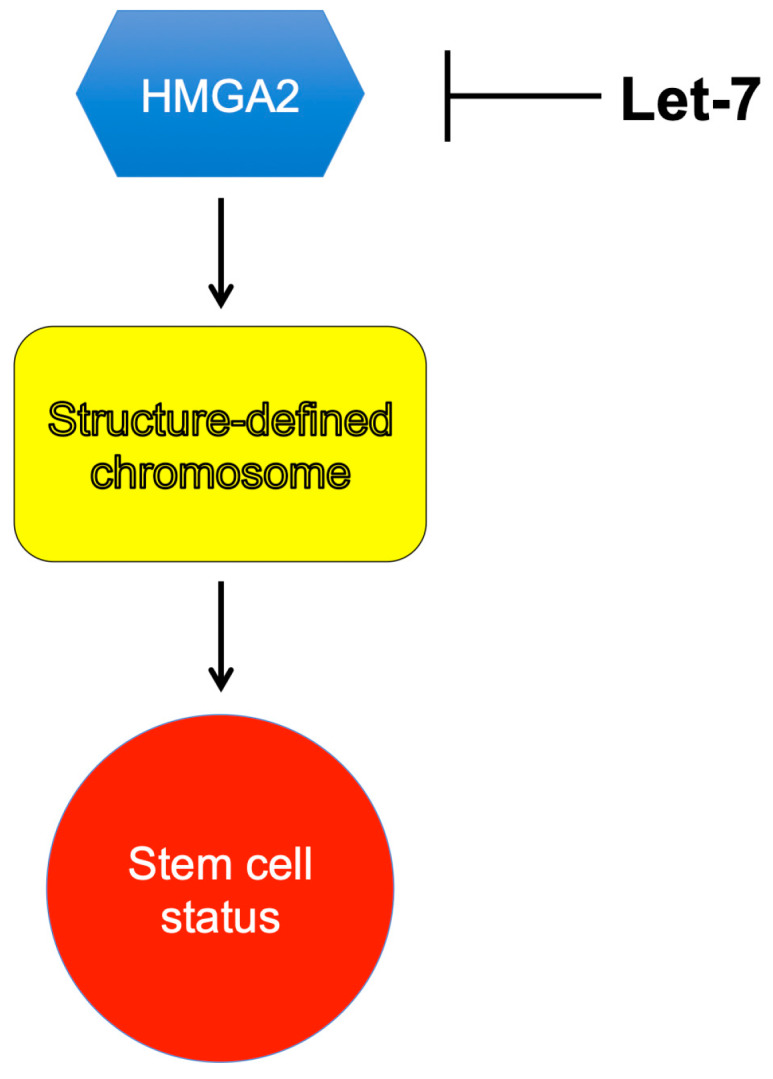
HMGA2 serves as an epigenetic or chromosome-remolding factor, to lock chromosome into a defined structure/conformation and to maintain the stem-cell status. MicroRNA let7 inhibits HMGA2 expression.

HMGA2 is a non-histone chromosome protein and has been linked to several phenotypic characteristics. Some of these phenotypes are reviewed here, except for its association with tumorigenesis. For the HMGA proteins’ role in tumorigenesis, please refer to review articles published in the past for details [112,113,114,115,116,117,118,119,120,121]. It looks likely that the main functions of HMGA2 are promoting cell proliferation and maintaining the stemness potency of stem cells. What is still obscure is the molecular mechanism behind these phenotypes and functions. We believe that HMGA2 is an epigenetic factor that programs or reprograms chromosomes into a “defined” state, to achieve these functions (Figure 3) [122,123]. This hypothesis is in contrast with the previous belief that HMGA2 serves as a transcriptional factor or an architecture/general transcriptional factor, to promote or inhibit transcription only. Recent evidence showed that HMGA2 might also affect other cellular processes, such as DNA replication. For instance, Droge and coworkers showed that HMGA2 protects stalled DNA replication forks and prevents the forks from collapsing, to enhance stem- and cancer-cell survival when these cells are challenged with DNA-replication stress [124]. This unique property may affect the sensitivity of cancer cells to chemotherapy drugs, especially topoisomerase poisons [125,126]. They estimated that about 10^5^ to 10^6^ molecules of HMGA2 exist in each human embryonic stem cell, which lead to one molecule of HMGA2 binding to 3 to 30 kb human chromosomal DNA or 10 to 100 nucleosome core particles (NCPs) on average [99]. Early studies by Goodwin and coworkers also showed that HMGA2 could compete with histone H1 for binding to nucleosomes [127]. A possible scenario is that the binding of HMGA2 to nucleosomes may “lock” chromosome into a specific state to allow the human embryonic stem cells to maintain their stemness status. Secondly, HMGA2 is regulated by microRNA let-7 [17,19,93]. Specifically, let-7b destabilizes HMGA2 mRNA by targeting the 3′ UTR [17,19,93]. As a result, HMGA2 expression is significantly reduced. Let-7 and HMGA2 play an important role in cell differentiation and should be considered as epigenetic factors.

Another poorly understood area is the stability of HMGA proteins, including HMGA2, in vivo. Cao et al. showed that HMGA2 could be SUMOylated in vitro and inside cells [128]. Ubiquitin-proteasome dependent degradation may be the pathway for HMGA2’s degradation [128,129]. Apparently, more studies are needed in this field. Furthermore, the function of the negatively charged C-terminal motif of HMGA2 is still unknown. Interestingly, all HMG proteins have a highly negatively charged C-terminus [130], indicating that the negatively charged C-terminus has important functions. The C-terminal motif of HMGA2 also contains several serine and threonine residues that can be phosphorylated by CK2. One immediate consequence of the negatively charged C-terminal motif and its phosphorylation is to regulate HMGA2’s binding to DNA and nucleosomes [47,48,50,51,52]. Nevertheless, more studies are needed.

As discussed above, HMGA2 plays an important role in adipogenesis and is an excellent target for the treatment of obesity [60]. Since the overexpression and/or aberrant-expression of HMGA2 is directly linked to the formation of a variety of malignant tumors, including lung cancer [131,132], breast cancer [133,134], prostate cancer [135], leukemia [136], and melanoma [137,138,139,140], HMGA2 appears to be an attractive target for anticancer drugs [117,141]. Several strategies may be used to target HMGA2 for therapeutic purposes. The first strategy is to target the AT-rich DNA-binding sequences that HMGA2 recognizes. For instance, we recently demonstrated that netropsin, a DNA minor groove binder, potently inhibits HMGA2 binding to DNA [89,141]. Intriguingly, netropsin strongly inhibited the differentiation of mouse pre-adipocyte 3T3-L1 cells into adipocytes. It is likely that the inhibition is accomplished through the inhibition of HMGA2 binding to the target DNA sequences during differentiation [89]. Other minor groove binders can also inhibit HMGA2 binding to DNA [89]. A disadvantage of this strategy is that netropsin non-specifically binds to any five AT base pairs and displays non-specific cytotoxicity to many cell types [142,143,144,145], which prevents it from becoming an effective anticancer and anti-obesity drug. Although it is possible to design a synthetic compound that targets a specific AT sequence [146,147,148], the lack of knowledge about what sequences HMGA2 recognizes inside a cell makes this strategy a mission impossible. The second strategy is to identify compounds that bind to HMGA2 and prevent it from binding to AT-rich DNA sequences. This is a tough job, because DNA-binding proteins (transcriptional factors) are considered “undruggable” due to the fact that they usually do not have enzymatic activities suitable for chemical intervention [149,150]. Additionally, HMGA2 is an IDP and lacks a deep pocket for ligand binding [20,32]. One way to overcome these difficulties is to develop an efficient method to identify inhibitors from existing small molecule repositories. Indeed, we established a medium-throughput screening method based on the protein–DNA interaction enzyme-linked immunosorbent assay (PDI-ELISA), to screen a small library containing 29 DNA-binding compounds, and successfully identified several small molecules that disrupt HMGA2 binding to the minor groove of AT-rich DNA sequences [89]. Recently, we developed a miniaturized automated AlphaScreen ultra-high-throughput screening (uHTS) assay to identify inhibitors targeting HMGA2-DNA interactions (unpublished results). After screening the LOPAC1280 compound library, we discovered several small molecule compounds that potently inhibit the HMGA2-DNA interaction through binding to HMGA2 (unpublished results). The third strategy is the use of the negatively charged C-terminus to inhibit HMGA2 binding to AT-rich DNA sequences. Recently, our unpublished results showed that the C-terminal motif of HMGA2 binds to the “AT-hook” DNA-binding motifs and inhibits HMGA2 binding to AT-rich DNA sequences. It is possible to synthesize the C-terminus mimics, to enhance the inhibition ability and also increase the stability in vivo [151,152]. The fourth strategy is to target HMGA2’s mRNA. Anti-sense oligomers and RNAi were used to lower the HMGA2 expression at the cellular level [18,41,153,154,155,156]. MicroRNA let-7b may also be used to decrease HMGA2 expression level. The delivery of these nucleic acids into cells or the target tissues may still be a challenge.

## Abbreviations

HMGA2	Mammalian high-mobility-group protein AT-hook 2
IDP	Intrinsically unstructured protein
d.p.c.	Days post-coitum
UTR	Untranslational region
GWA	Genome-wide association
SNP	Single nucleotide polymorphism
NSC	Neural stem cell
HSC	Hematopoietic stem cell
MSC	Mesenchymal stem cell
PPARγ	Peroxisome proliferator-activated receptor γ
NCP	Nucleosome core particle
CK2	Casein kinase 2
PDI-ELISA	Protein–DNA interaction enzyme-linked immunosorbent assay
HTS	High-throughput screening
